# Influence of Microbiota on Viral Infections

**DOI:** 10.1371/journal.ppat.1002681

**Published:** 2012-05-17

**Authors:** Jessica Wilks, Tatyana Golovkina

**Affiliations:** Department of Microbiology, University of Chicago, Chicago, Illinois, United States; University of Florida, United States of America

Most pathogens gain access to the host through surfaces of the body that are exposed to the surrounding environment and rife with resident microorganisms, termed microbiota. Microbiota play an integral role in modulating host health. One significant benefit of the microbiota is that they provide protection against incoming bacterial pathogens [1]. Commensals make their immediate environment inhospitable to many pathogens by producing biosurfactants, by competing for sites of attachment and nutrients, and by excreting metabolites with antimicrobial effects [1]. Furthermore, the presence of commensals promotes maturation of secondary lymphoid organs in the intestine, which are the first line of defense in the intestinal mucosa [2]. Therefore, when a pathogen infiltrates the host, it is not entering a sterile environment, but one that has been shaped by a dynamic commensal community. Although many interactions between bacterial pathogens and the microbiota have been characterized [1], little is known about the interplay between viral pathogens and the natural flora of the host. Are viral pathogens blind to the commensal microbes surrounding them? Judging from recent publications, this appears not to be the case. There is strong evidence that the microbiota can either protect the host from virally induced disease or promote viral propagation/transmission, through direct or indirect mechanisms.

## Beneficial Influence of Microbiota on Antiviral Immunity

**Figure 1:**
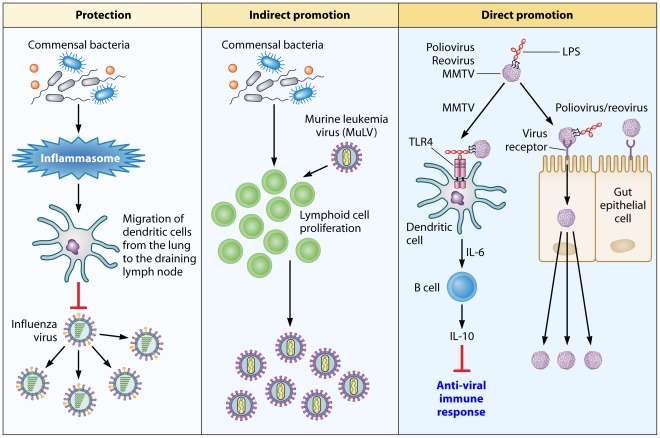
An overview of how the commensal microbiota influence viral pathogenesis. Protection: The microbiota of the host activates the inflammasome by priming signal 1 for IL-1β and IL-18 secretion. The secretion of these cytokines induces migration of dendritic cells from the lung to the draining lymph node, where they prime T cells. The downstream effect of T-cell priming is protection of the host against influenza virus-induced pathology. Indirect promotion: In the case of MuLV, the microbiota of the host stimulate proliferation of lymphoid cells that are targeted by the virus. Direct promotion: Microbial ligands, such as LPS, are utilized by viruses to enhance their attachment to target cells (polio virus and reovirus) or to counteract the antivirus immune response by activating the TLR4 pathway, which leads to IL-10 production (MMTV).

Because the microbiota are present at the sites used by viruses to gain entry to their host, they can potentially alter the outcome of infection. For example, the commensal microbiota of the insect vector *Aedes aegypti* indirectly mitigate Dengue virus transmission [3]. Mosquitoes whose commensals are ablated by antibiotics have higher viral titers than those that are left untreated. Moreover, mosquitoes possessing their natural flora show elevated expression of several immune-related genes, including those encoding antimicrobial peptides regulated by Toll-like receptor (TLR) pathways [3]. The authors hypothesize that the endogenous bacterial flora stimulate the mosquitoes' antiviral immune system through basal-level activation of innate immune pathways. Likewise, ablation of the natural flora of mice via antibiotic treatment increases the animals susceptibility to influenza A virus (Figure 1). Again, the mechanism of microbiota-mediated protection against the virus appears to be indirect—the microbiota are responsible for activation of the inflammasome [4], which is required for defense against influenza [5]. Inflammasome activation induces migration of dendritic cells from the lung to the draining lymph node, to prime influenza-specific T-cell responses [4]. Interestingly, a TLR agonist such as lipopolysaccharide (LPS) added intranasally or intrarectally restores the immune response to influenza in antibiotic-treated animals [4].

It should be stressed that both of the aforementioned studies used antibiotics to alter the microbiota of the host. However, antibiotic treatment can lead to changes in the host's physiology that are independent of microbiota disturbance. Additionally, antibiotic treatment may not eliminate all microorganisms from the host—many microbes are known to be resistant to antimicrobial therapies [6], and the majority of commensal species are unculturable, making it difficult to prove the existence of an antibiotic-induced sterile environment [7]. To subvert this problem, one can use germ-free (GF) organisms. These organisms are completely sterile, and exhibit normal developmental patterns overall. However, both the gut associated lymphoid tissue and the intestinal immune cells of these animals are underdeveloped. Consequently, when studying the interaction between the host's microbiota and a given pathogen, it is imperative to use both antibiotic-treated and GF animals to account for the limitations of both experimental systems.

## Indirect Promotion of Viral Infections by Commensal Microbiota

Although microbiota can help the host fight viral infections, as in the case of influenza, it may also enhance viral infection, either indirectly or directly. One example of the indirect beneficial effects of microbiota on virus replication is the promotion of viral infection by stimulating the proliferation or activation of target cells (Figure 1). This is particularly true of retroviruses that target proliferating cells. For example, GF mice infected with murine leukemia virus (MuLV) are relatively resistant to virally induced leukemia compared to conventionally housed or specific pathogen free (SPF) mice [8], [9]. Immunization of MuLV-infected GF mice with sheep red blood cells results in a significant increase in leukemia development comparable to that of infected SPF mice [9]. The authors hypothesize that the decrease of MuLV pathogenicity in GF mice could be due to microbiota-stimulated division of lymphoid cells, which would cause an increase in virus replication and, thus, a higher frequency of leukemia. It should be noted that other studies demonstrate that GF mice are more susceptible than SPF mice to MuLV-induced leukemia [10], which conflicts with the aforementioned findings. One potential explanation for this discrepancy is that the studies showing increased susceptibility of GF mice to MuLV were conducted before it was revealed that some MuLV isolates contain a contaminating lactate dehydrogenase-elevating virus (LDV). LDV induces systemic lymphocyte activation [11] and could have skewed the results of the investigations.

## Direct Assistance of Viral Infections by Microbiota

To date, two studies, including our own, indicate that viruses from three distinct families rely on commensal organisms for efficient replication/transmission [12], [13]. In the first study, Kuss et al. found that antibiotic-treated, poliovirus-susceptible mice show lower mortality following oral poliovirus infection compared to untreated mice (Figure 1). Importantly, replication of the virus in the mouse intestines is dependent on the microbiota, as GF or antibiotic-treated mice secrete poorly infectious virus. Gram-negative or Gram-positive bacteria incubated with poliovirus greatly promote virus infectivity in tissue culture cells. This enhancement did not require live bacteria, as bacterial surface polysaccharides, including LPS and peptidoglycan (PG), have the same effect on virus infectivity [12]. Importantly, these findings were not unique to poliovirus; the pathogenesis of reovirus, an unrelated enteric virus, is also more severe in the presence of intestinal microbes [12].

We discovered that Mouse Mammary Tumor Virus (MMTV), a retrovirus transmitted through the milk, utilizes the innate immune Toll-like receptor TLR4 to induce tolerance to itself, and thus to evade the antiviral response (Figure 1) [13]. Triggering of TLR4 by the virus results in interleukin 6 (IL-6)-mediated production of the immunosuppressive cytokine IL-10, which is required for blockage of the antiviral response [13]. MMTV does not signal directly through TLR4 but uses LPS, a well-characterized TLR4 ligand, to trigger the receptor, as LPS-free MMTV stocks fail to induce IL-10 production. Furthermore, GF mice infected with MMTV by intraperitoneal injection are unable to transmit infectious virus to their offspring. Therefore, MMTV exploits tolerogenic properties of commensal bacteria to induce unresponsiveness to itself. Together, the two studies reveal that orally transmitted viruses from three diverse families take advantage of the gut microbiota for successful propagation. Exploitation of the microbiota of the host can now be added to the list of innovative evasion strategies used by viruses.

## Do Lentiviruses Utilize Microbiota for Their Benefit?

Like the viruses described in the preceding section, HIV-1 is also transmitted across mucosal surfaces, which are rich in microbiota. This prompts the question—do the microbiota contribute to infection with HIV-1? People chronically infected with HIV exhibit raised plasma levels of LPS [14]. Moreover, the peptide derived from the V3 loop of gp120 specifically interacts with the lipid A moiety of LPS, as does the full gp120 protein [15]. In addition, glycerol monolaurate, a widely used antimicrobial compound, protects rhesus macaques from acute infection of simian immunodeficiency virus (SIV) [16]. Therefore, it is possible that HIV and SIV may also take advantage of commensal bacteria to assure successful propagation and spread.

## Concluding Remarks

With the advent of the Human Microbiome Project, we are now aware of the number and diversity of microbes that make the human body their primary place of residence. Consequently, the microbiota can no longer be ignored when studying host–pathogen interactions. The influences of microbiota on virus infections could be either protective or detrimental for the host. Whereas the microbiota positively regulate adaptive immune responses against influenza, they suppress antivirus adaptive responses against MMTV and facilitate replication of poliovirus and reovirus by enhancing virus attachment to target cells. Thus, microbiota play a dual role in virus–host interactions. An open question that currently drives research related to microbiota is how the microbiota can be manipulated so that the host is protected from deleterious infections. In the case of pathogens that take advantage of the microbiota, one can hope to find a way to ablate these interactions, thus preventing pathogen spread/propagation. This could be done either by manipulating the composition of the microbiota (ablation of a specific microbe exploited by a virus) or by blocking interactions between the viral pathogen and specific bacterial compounds that benefit the pathogen. Future discoveries in the area of microbiota–pathogen interactions will undoubtedly unveil new opportunities for therapeutic interventions in infectious disease.
